# Supplementary material for: Association between long COVID and vaccination: A 12-month follow-up study in a low- to middle-income country
Source: PLoS One. 2023 Nov 22;18(11):e0294780. doi: 10.1371/journal.pone.0294780 (PMC10664948; doi:10.1371/journal.pone.0294780)
Supplement: S1 File — (DOCX) [file pone.0294780.s002.docx]

Informed Consent

**Topic: Long-term Symptoms, Functional and adverse outcomes in COVID-19 survivors; in the follow-up of 1 year**

**Principal Investigator: Dr Madiha Ismail/ Dr Samar Fatima**

I am ______________________ and I work at The Aga Khan University. I would like to invite you to take part in this study titled “Long-term Symptoms, Functional and adverse outcomes in COVID-19 survivors; in the follow-up of 1 year”. If you want any further explanations or knowledge about any questions, feel free to ask research team member. Please make sure that your have been satisfactorily answered before consenting to participate in this study.

**1. PURPOSE OF THIS RESEARCH STUDY**

In this study we are exploring Long-term Symptoms, Functional and adverse outcomes in COVID-19 survivors. You have been asked to participate because according to our record you recovered from COVID-19 last year.

**2. PROCEDURES**

In this study, we will collect specific details about your history,­­­­­­­­­­­­­­­­­­ and laboratory parameters from your medical records. In addition, we are doing a telephonic survey, and we will ask you few questions regarding your illness and present health status. The survey will take approximately 25-30 minutes of your time.

**3. POSSIBLE RISKS OR DISCOMFORT**

Besides giving up your valuable time, there will be no risks or costs associated with taking part in this study. In case of Emergency visit treatment of your patient will be carried out as usual.

**4. POSSIBLE BENEFITS**

On participating there may be no direct benefit to you and your patient. However, the results of this study will help improve the management and discharge planning of patients in future.

**6. CONFIDENTIALITY**

All information collected for this study will be kept confidential and only PI and team members will have access. We will not record any specific information about you such as your name or address. The results of the study will be published collectively; Individual identity and record will be kept confidential according to rules and regulations.

**7. VOLUNTARY PARTICIPATION:**

You are free to choose whether or not to participate in this study and have a right to leave study at any stage. There will be no penalty or loss of benefits to which you are otherwise entitled if you choose not to participate.

**8. AVAILABLE SOURCES OF INFORMATION**

If you want to ask any further questions you can contact principal investigator of this study: Dr Madiha Ismail at 021-3486 1146 between 9am -5 pm

**9. AUTHORIZATION**

I have read and understand this consent form, and I volunteer to participate in this research study. I have been explained in detail regarding the purpose, procedures, possible benefits and risk or discomforts of this study, and given opportunity to fully understand the consent form. I will be provided a copy of consent form.

Are you agreeing to participate in this study: Yes No

Name of person obtaining consent: _________________

Signature of person obtaining consent: _________________

Name of witness: _________________

Signature of witness: _________________

Date: _____________________________

If No:

Reason for not consenting to participate in the study: _________________________________________________________________________________

_________________________________________________________________________________
