# Supplementary material for: Association between long COVID and vaccination: A 12-month follow-up study in a low- to middle-income country
Source: PLoS One. 2023 Nov 22;18(11):e0294780. doi: 10.1371/journal.pone.0294780 (PMC10664948; doi:10.1371/journal.pone.0294780)
Supplement: S2 File — (DOCX) [file pone.0294780.s003.docx]

**Table S1: COVID-19 survivors’ health-related characteristics stratified according to vaccination status.**

| **Characteristics of disease on index admission based on Vaccination status, n=474** | | | | |
| --- | --- | --- | --- | --- |
|  | **Fully vaccinated % (n=90)** | **Partially vaccinated % (n=91)** | **Unvaccinated % (n=293)** | **p-value** |
|  |  |  |  |  |
| **Disease Severity** |  |  |  |  |
| Non-Severe | 45.6 (41) | 37.4 (34) | 31.8 (93) | 0.052 |
| Severe/Critical | 54.4 (49) | 62.6 (57) | 68.2 (200) |  |
| Invasive mechanical ventilation | 0 | 2.2 (2) | 4.44 (13) | 0.077 |
| Readmission | 15.6 (14) | 12.2 (11) | 21.51 (63) | 0.056 |
| Pulmonary complications^$^ | 13.4 (12) | 17.6 (16) | 24.2 (71) | 0.023 |
| **Health-related characteristics on follow-up stratified on vaccination, n=452** | | | | |
|  | **Fully vaccinated n=87** | **Partially vaccinated**  **n= 88** | **Unvaccinated n=277** | **p-value** |
| Asymptomatic | 80.5 (70) | 79.5 (70) | 62.1 (172) | <0.000 |
| New/worsened/persistent symptoms related to Long COVID | 19.5 (17) | 20.5 (18) | 37.9 (105) | 0.000 |
| Fatigue (n=124) | 17.2 (15) | 17.1 (15) | 33.9 (94) | 0.000 |
| Minimal (n=42) | 11.9 (5) | 14.8 (13) | 8.6 (24) |  |
| Shortness of breath (SOB)/ chest tightness/ wheezing | 14.9 (13) | 19.1 (17) | 30 (84) | 0.005 |
| Cough (n= 72) | 3.4 (3) | 12.5% (11) | 20.9 (58) | 0.000 |
| Difficulty ambulating due to breathlessness | 9.2 (8) | 6.7 (6) | 15.8 (44) | 0.04 |
| Feverish | 1.1 (1) | 3.4 (3) | 8.3 (23) | 0.025 |
| Continued loss of taste and/or smell | 2.3 (2) | 1.1 (1) | 2.8 (8) | 0.83 |

**Table S2: COVID-19 survivors’ return to work and HADS score stratified according to vaccination status.**

|  | **Fully vaccinated % (n=87)** | **Partially vaccinated**  **% (n= 88)** | **Unvaccinated % (n=277)** | **p-value** |
| --- | --- | --- | --- | --- |
| Return to Employment (n=266, NA=193) | n=40 | n=47 | n=176 |  |
| Yes (202) | 87.5 (35) | 95.7 (45) | 69.4 (122) | 0.000 |
| No (61) | 12.5 (5) | 4.3 (2) | 30.7 (54) |  |
| Able to return to work in 60 days following discharge. | n=36 | n=44 | n=131 |  |
| Yes | 94.4 (34) | 90.0 (40) | 84 (110) | 0.177 |
| No | 5.6 (2) | 3.4 (6) | 16 (21) |  |
| Anxiety (HADS-A score >11) |  |  |  |  |
| n =16 | 8 (7) | 3.4(3) | 2.1(6) | 0.035 |
| Depression (HADS-D score >11) |  |  |  |  |
| n =20 | 4.6 (4) | 1.1 (1) | 5.4 (15) | 0.246 |
| Combined anxiety and depression (HADS-A and/or HADS-D score >11) (28) |  |  |  |  |
|  | 8 (7) | 3.4 (3) | 6.4 (18) | 0.42 |
