# Supplementary material for: Association between long COVID and vaccination: A 12-month follow-up study in a low- to middle-income country
Source: PLoS One. 2023 Nov 22;18(11):e0294780. doi: 10.1371/journal.pone.0294780 (PMC10664948; doi:10.1371/journal.pone.0294780)
Supplement: Attachment — Submitted filename: Response to Reviewers.docx [file pone.0294780.s004.docx]

**Response to Reviewer**

We would like to express our sincere gratitude for considering our manuscript, titled "The Post-Pandemic Aftermath in a LMIC: A 12-month follow-up study on Long COVID symptoms and the impact of vaccination in hospitalized patients," for review at PLOS ONE. We greatly appreciate the time and effort put forth by both you and the reviewers in evaluating our work. In response to the insightful comments provided by the reviewers, we have diligently revised and modified our manuscript. Every suggestion made by the reviewers has been incorporated into the manuscript to enhance its quality and clarity.

With these revisions, we believe that our manuscript is now well-aligned with the high standards of PLOS ONE and is suitable for publication. We eagerly await your feedback on the revised submission and are ready to address any further questions or comments you may have.

Yours sincerely,

On behalf of all the authors,

Dr. Madiha Ismail

**Journal Requirements:**

1. Please ensure that your manuscript meets PLOS ONE's style requirements, including those for file naming. The PLOS ONE style templates can be found at 
<https://journals.plos.org/plosone/s/file?id=wjVg/PLOSOne_formatting_sample_main_body.pdf> and 
<https://journals.plos.org/plosone/s/file?id=ba62/PLOSOne_formatting_sample_title_authors_affiliations.pdf>

2. We suggest you thoroughly copyedit your manuscript for language usage, spelling, and grammar. If you do not know anyone who can help you do this, you may wish to consider employing a professional scientific editing service.

Upon resubmission, please provide the following: 
● The name of the colleague or the details of the professional service that edited your manuscript
● A copy of your manuscript showing your changes by either highlighting them or using track changes (uploaded as a *supporting information* file)
● A clean copy of the edited manuscript (uploaded as the new *manuscript* file)

3. Please include captions for your Supporting Information files at the end of your manuscript, and update any in-text citations to match accordingly. Please see our Supporting Information guidelines for more information: <http://journals.plos.org/plosone/s/supporting-information>.

**Reply:**

●We have made corrections according to PLOS ONE's style requirements and also copyedit the manuscript for language usage, spelling, and grammar.

● The name of the colleague that has edited our manuscript is Akbar Shoukat Ali (Research Associate, Department of Medicine, Aga Khan University Hospital, Karachi, Pakistan)
● A copy of the revised manuscript showing track changes has been uploaded as a *supporting information* file)
● A clean copy of the edited manuscript has been uploaded as the new *manuscript* file

**"The following represents our response to the reviewers' comments."**

**Reviewer 1**

**Comment 1:**

Severe/critical disease was present in 64% of the population, and 25.6% required non-invasive/invasive mechanical ventilation.
64% of the 481 patients analysed, right? Please write numbers along with percentage
**Reply:**

We have added numbers with the percentages as per your recommendation. This has also been corrected and highlighted in the manuscript. For further clarification, we have also added here. Also in the abstract, we have a limitation of a word count of 300.

Severe/critical disease was present in 64% (312/481) of the population, and 25.6% (120/481) required non-invasive/invasive mechanical ventilation.

**Comment 2:**

The authors mention that ,”On Long COVID, to the best of our, knowledge, prevalence data, estimates of the at-risk population, and the burden have not been reported from Pakistan”. However there is a study , “Analysing the psychosocial and health impacts of Long COVID in Pakistan: A cross sectional study” from AGKU by Madeeha Khan et al. Please clarify this and highlight the differences between the two studies.

**Reply :**

Thank you for bringing this study to our attention. We did not include this study in our manuscript because this is a pre-print version. Additionally, at the time of manuscript preparation, this preprint was not available and it was published online on May 23, 2023. The participants included in the study done by Madeeha Khan et al. differ from ours and we are mentioning the differences here and also have included this in the discussion section of the manuscript. (doi: https://doi.org/10.1101/2023.05.22.23290323)

1) In our study we looked for Long COVID symptoms in patients who were hospitalized between February 1, 2021, and June 7, 2021, and interviewed them for Long COVID one year after their discharge from the hospital, while in the study by Madeeha Khan et al., only 8.4% (n=13) required hospitalization and most of participant included were outpatient. Moreover, only a small proportion (n=9; 16.7%) of these long haulers were hospitalized during the acute phase.
2) The author evaluated Long COVID symptoms at 12 weeks following infection from COVID-19 disease, as compared to our study in which we interviewed the patients one year after their discharge from the hospital.
3) Madeeha Khan et al. assessed the health symptoms as well as psychological impact but did not analyze it based on the severity of the disease and vaccination status.
4) Among the 155 COVID-19-positive patients, 54 (35%) continued to experience symptoms for more than 12 weeks after infection, compared to 29.9% of our patients at the one-year mark.
5) The most frequent symptoms in the Madeeha et al study were muscle problems and fatigue (14.7%) followed by breathing difficulties such as breathlessness, dyspnea, painful breathing, or cough (12.6%). However, in our study, this percentage was higher with fatigue reported at 26.2% and symptoms like shortness of breath, chest tightness, and wheezing at 24.1%. The elevated occurrence of this disparity within our patient population may stem from the fact that a substantial majority of our patients, comprising 64% (312 out of 481), suffered from severe or critical disease. Moreover, all our patients were hospitalized.
6) The author used different scales for assessing stress, anxiety and depression as compared to our study.
7) We only included patients with confirmed positive SARS-CoV-2 RT-PCR. However, in their study, among 300 patients, 155 (51.7%) had COVID-19 either confirmed by positive RT-PCR and/or diagnosed by a general physician on clinical grounds without any testing.

**Comment 3:**

As per the above study (by Madeeha Khan et al.), A small proportion (n=9; 16.7%) of these long haulers were hospitalized during the acute phase. In other studies too, symptoms of long COVID have been observed in patients who had mild symptoms, in contrast to your study where most had severe symptoms.

**Reply :**

In our study as well, patients with non-severe COVID suffered from long COVID symptoms, however as compared to severe/critical disease, individuals with severe/critical disease were more likely to have long COVID. This observation aligns with the findings of Xue Zhang et al. (https://www.ncbi.nlm.nih.gov/pmc/articles/PMC8482055/).

Furthermore, we only included patients necessitating hospitalization and testing positive through RT-PCR or Antigen tests. Given that patients with mild disease typically do not require hospitalization, our study naturally demonstrated a lower proportion of such cases in comparison. Madeeha Khan et al reported that only 13 (8.4%) in their study population had required hospitalization.

**Comment 4:**

Expedited NOT expeditated

**Reply :**

We have corrected the spelling error.

**Comment 5:**

Low antibody titres in patients with COVID-19 have been reported to be predictive of Long COVID regardless of hospitalization status[10], and Perlis et al from USA reported that full vaccination resulted in a lower risk for Long COVID with OR 0.72[11]
Suggest rephrasing to ..Low antibody titres to SARSCoV 2 in patients………….

**Reply :**

This has been rephrased to:

It has been observed that low antibody titres to SARS‑CoV‑2 have been associated with a greater likelihood of experiencing Long COVID, regardless of hospitalization status. Perlis et al. conducted a study and observed that full vaccination resulted in a lower risk of developing long COVID with an OR of 0.72.

**Comment 6:**

The abstract mentions the study period as between 1st January 21 to 30th June 21, whereas the article mentions it as 1st Feb 21 to 7th June 21.

**Reply :**

This has been corrected and highlighted in the manuscript. The study recruited patients who were admitted between February 1, 2021, and June 7, 2021, and it has been rechecked from the data.

**Comment 7:**

COVID-19 is known to unmask pre-existing diabetes and also cause thyroid disease, the symptoms of which may resemble those of Long COVID. Were patients asked for new onset diabetes or hypothyroidism, following COVID 19 ?

**Reply :**

Thank you for bringing this to our attention. It is an area of interest for us too. However, these could not be assessed in our study as these diagnoses ( new onset diabetes or hypothyroidism) would have required a review of the diagnostic labs/criteria. We will mention this in our limitation section.

**Comment 8:**

Grammatical errors need to be addressed.

**Reply :**

We have corrected the grammatical errors and the manuscript has been revised.

**Reviewer 2**

**Comment 1:**

Page 1, Title, “The Post-Pandemic Aftermath in a LMIC: A 12-month follow-up study on Long COVID symptoms and the impact of vaccination in hospitalized patients”: This title seems vague. The authors may consider rephrasing to “Association between vaccination status and occurrence of long COVID symptoms after hospitalization for COVID-19 in a low- or middle-income country”.

**Reply :**

We appreciate your input and agree that clarity in the title is important. We have considered your suggestion, and our revised title is now:

Association between Long COVID and Vaccination: A 12-Month Follow-up Study in a Low- to Middle-Income Country

**Comment 2 :**

In accordance with my first comment, the authors **may wish to reconstruct their manuscript focusing on the association between vaccination status and long COVID**. Although there are studies on this subject from high-income countries (summarized in Byambasuren O et al. BMJMed 2023), data from low- or middle-income countries (LMIC) are scarce and useful.

**Reply :**

In response to the suggestion provided in your first comment, we appreciate the emphasis on exploring the association between vaccination status and long-term COVID. It is indeed a valuable point that data from low- or middle-income countries (LMIC) are limited in this context. On your advice, we have reconstructed our manuscript with more focus on the association between vaccination status and long COVID.

However, in our study, we aimed to address not only the association between long COVID and vaccination but also its relationship with the severity of the disease. This comprehensive approach allows us to provide a more holistic understanding of Long COVID in our specific population.

**Comment 3:**

If the authors choose to follow the above suggestion, then they should reformat their Tables. For example, Table 1 and Table 3 could present baseline characteristics and long COVID symptoms of COVID-19 survivors according to vaccination status (i.**e., non-vaccinated, partially vaccinated and fully vaccinated).**

**Reply :**

Thank you for your suggestion. Initially, we had intended to follow the same approach. However, due to a disparity in the total population number between the baseline characteristics (n=481) and the total number of patients for whom vaccination status was available (n=474, with Long COVID symptoms assessed in 452 patient-survivors), we decided to create separate tables, one for baseline characteristics and another for vaccination. This choice was made to prevent any potential confusion during the reading of our manuscript as well as in the analysis. Additionally, we aimed to assess Long COVID based on its severity.

Furthermore, for chi-square testing and calculation of the odd ratio, we needed two categories, so we opted for vaccinated (partially/fully) and unvaccinated groups.

However, recognizing the merit of your insightful suggestion, we have taken it into account and have included a comprehensive table detailing the disparities in outcomes among partially vaccinated, fully vaccinated, and unvaccinated individuals in the supplementary file. We are also including these tables in this document.

**S1 Table: COVID-19 survivors’ health-related characteristics stratified according to vaccination status.**

| **Characteristics of disease on index admission based on Vaccination status, n=474** | | | | |
| --- | --- | --- | --- | --- |
|  | **Fully vaccinated % (n=90)** | **Partially vaccinated % (n=91)** | **Unvaccinated % (n=293)** | **p-value** |
|  |  |  |  |  |
| **Disease Severity** |  |  |  |  |
| Non-Severe | 45.6 (41) | 37.4 (34) | 31.8 (93) | 0.052 |
| Severe/Critical | 54.4 (49) | 62.6 (57) | 68.2 (200) |  |
| Invasive mechanical ventilation | 0 | 2.2 (2) | 4.44 (13) | 0.077 |
| Readmission | 15.6 (14) | 12.2 (11) | 21.51 (63) | 0.056 |
| Pulmonary complications^$^ | 13.4 (12) | 17.6 (16) | 24.2 (71) | 0.023 |
| **Health-related characteristics on follow-up stratified on vaccination, n=452** | | | | |
|  | **Fully vaccinated n=87** | **Partially vaccinated**  **n= 88** | **Unvaccinated n=277** | **p-value** |
| Asymptomatic | 80.5 (70) | 79.5 (70) | 62.1 (172) | <0.000 |
| New/worsened/persistent symptoms related to Long COVID | 19.5 (17) | 20.5 (18) | 37.9 (105) | 0.000 |
| Fatigue (n=124) | 17.2 (15) | 17.1 (15) | 33.9 (94) | 0.000 |
| Minimal (n=42) | 11.9 (5) | 14.8 (13) | 8.6 (24) |  |
| Shortness of breath (SOB)/ chest tightness/ wheezing | 14.9 (13) | 19.1 (17) | 30 (84) | 0.005 |
| Cough (n= 72) | 3.4 (3) | 12.5% (11) | 20.9 (58) | 0.000 |
| Difficulty ambulating due to breathlessness | 9.2 (8) | 6.7 (6) | 15.8 (44) | 0.04 |
| Feverish | 1.1 (1) | 3.4 (3) | 8.3 (23) | 0.025 |
| Continued loss of taste and/or smell | 2.3 (2) | 1.1 (1) | 2.8 (8) | 0.83 |

**S2 Table: COVID-19 survivors’ return to work and HADS score stratified according to vaccination status.**

|  | **Fully vaccinated % (n=87)** | **Partially vaccinated**  **% (n= 88)** | **Unvaccinated % (n=277)** | **p-value** |
| --- | --- | --- | --- | --- |
| Return to Employment (n=266, NA=193) | n=40 | n=47 | n=176 |  |
| Yes (202) | 87.5 (35) | 95.7 (45) | 69.4 (122) | 0.000 |
| No (61) | 12.5 (5) | 4.3 (2) | 30.7 (54) |  |
| Able to return to work in 60 days following discharge. | n=36 | n=44 | n=131 |  |
| Yes | 94.4 (34) | 90.0 (40) | 84 (110) | 0.177 |
| No | 5.6 (2) | 3.4 (6) | 16 (21) |  |
| Anxiety (HADS-A score >11) |  |  |  |  |
| n =16 | 8 (7) | 3.4(3) | 2.1(6) | 0.035 |
| Depression (HADS-D score >11) |  |  |  |  |
| n =20 | 4.6 (4) | 1.1 (1) | 5.4 (15) | 0.246 |
| Combined anxiety and depression (HADS-A and/or HADS-D score >11) (28) |  |  |  |  |
|  | 8 (7) | 3.4 (3) | 6.4 (18) | 0.42 |

**Comment 4:**

Following the above comment, the categorization into three groups (i.e., non-vaccinated, partially vaccinated and fully vaccinated) may be more informative than the current categorization (Tables 5-6) into two groups (i.e., non-vaccinated versus partially/fully vaccinated).

**Reply :**

This table has been added as a supplementary file.

**Comment 5:**

This categorization into three groups could be applied in the multivariate analysis exploring factors associated with long COVID (Table 6 of page 19). It would be nice to see whether there is a “dose-dependent” effect; i.e., is the odds ratio for the association between full vaccination status and long COVID nominally smaller than the odds ratio for the association between partial vaccination status and long COVID?

**Reply :**

As per your advice, this has been changed.

On multivariable analysis, after adjusting for age, gender, presence of co-morbid conditions and disease severity, vaccination status was found to be an independent predictor of Long COVID with an Odds ratio of 2.42(95% CI 1.52-3.84) for the unvaccinated cohort. Fully vaccinated and partially vaccinated patients had 62% and 56% reduced risk of developing Long COVID respectively. See **Table 7.**

**Table 7: Multivariate analysis for factors associated with Long COVID**

| **Variables** | **adjusted Odds ratio (95% Confidence interval)** | **p-value** |
| --- | --- | --- |
| **Female gender** | 1.14 (0.76-1.70) | 0.654 |
| **Age > 60 years** | 2.62(1.70-4.04) | 0.000 |
| **>2 comorbid conditions** | 1.24(0.70-2.21) | 0.463 |
| **Severe Disease** | 1.84(1.13-2.97) | 0.014 |
| **Fully vaccinated** | 0.38 (0.20-0.7) | 0.002 |
| **Partially vaccinated** | 0.44(0.24-0.80) | 0.007 |

*aOR(adjusted odd ratio) for unvaccinated 2.42(1.52-3.84)*

**Comment 6:**

In general, language editing may substantially benefit the manuscript. In specific, the authors may wish to use short sentences. Take for example, on page 2, Abstract, the long sentence: “On Long COVID, to the best of our, knowledge, prevalence data, estimates of the at-risk population, and the burden have not been reported from Pakistan, due to absence of prospective longitudinal studies on COVID-19 patients”.

**Reply :**

We appreciate your suggestion regarding language editing. Ensuring the manuscript's language and presentation meet the highest standards is crucial. We have worked on improving the manuscript's overall language and readability to enhance its clarity and impact

We have shortened the sentence and changed it in the manuscript accordingly:
As of now, there is a lack of prevalence data, estimates regarding at-risk populations, or any burden associated with Long COVID in Pakistan. This gap in knowledge primarily results from the absence of prospective longitudinal studies in the region.

**Comment 7:**

Page 3, Abstract: In the Results paragraph, it is written that “The most prominent symptoms that persisted even one year after infection…”. However, in the Conclusions paragraph, it is written that “This study reports the long-term health outcomes of COVID-19 survivors at 1-year following hospital discharge…”. The authors may wish to clarify when they assessed the study outcome; at one year after infection or one year after hospital discharge

**Reply :**

This has been rephrased in the manuscript, we did the study one year after discharge from the hospital.

**Comment 8:**

Page 6, Eligibility criteria and data collection, “Adult patients admitted between February 1, 2021, and June 7, 2021…”: In page 2, Abstract, it was written “…recruited patients aged > 18 years who were admitted between 1st January and June 30, 2021”. The authors may wish to correct such inconsistencies throughout their manuscript.

**Reply :**

We apologize for this error, this has been corrected in the manuscript.

**Comment 9:**

There are two Tables 6; one in page 18 and one in page 19.

**Reply :**

This has been corrected.

**Comment 10:**

Page 19, Figure 2 could be polished. For example, the authors could delete the “Chart title”.

**Reply :**

This has been changed.

**Comment 11:**

Pages 20-27: Discussion could be substantially shortened.

**Reply :**

As per your suggestion, we have shortened the discussion, from around 2416 words, we have reduced the discussion to 1995 words(pages 21-26)
