# Supplementary material for: Association between long COVID and vaccination: A 12-month follow-up study in a low- to middle-income country
Source: PLoS One. 2023 Nov 22;18(11):e0294780. doi: 10.1371/journal.pone.0294780 (PMC10664948; doi:10.1371/journal.pone.0294780)
Supplement: Attachment — Submitted filename: Response to Reviewers 2.docx [file pone.0294780.s005.docx]

**Response to Reviewer**

Thank you for your feedback on our revised manuscript (PONE-D-23-22756R1), titled “Association between Long COVID and Vaccination: A 12-Month Follow-up Study in a Low- to Middle-Income Country” at PLOS ONE. We are pleased to address the queries raised by Reviewer 1 and are equally delighted to learn that our response has met the satisfaction of Reviewer 2.

In response to Reviewer 1's thoughtful input, we have diligently refined our manuscript. These revisions have been made with great care to align our work with PLOS ONE's stringent publication standards. We eagerly await your assessment of the revised submission and remain at your disposal for any further questions or comments.

Yours sincerely,

On behalf of all the authors,

Dr. Madiha Ismail

**Journal Requirements:**

1. Please review your reference list to ensure that it is complete and correct. If you have cited papers that have been retracted, please include the rationale for doing so in the manuscript text, or remove these references and replace them with relevant current references. Any changes to the reference list should be mentioned in the rebuttal letter that accompanies your revised manuscript. If you need to cite a retracted article, indicate the article’s retracted status in the References list and also include a citation and full reference for the retraction notice.

2. Additional Editor Comments:

There are some clarifications and edits that are requested, please address.

**Reply:**

●	In response to your request for a review of our reference list to ensure its completeness and accuracy, we have thoroughly examined the references cited in our manuscript. We have checked and found that there were no retracted papers in our reference list. However, we have removed 2 references as we had to reduce the discussion as per the reviewer's advice.

- Sure we are providing the answer to the queries raised by Reviewer 1

**"The following represents our response to the reviewers' comments."**

**Reviewer 1**

**Comment 1:**

Were long COVID symptoms the reason for re-admission in any of the readmitted patients?

**Reply:**

We would like to express our gratitude to the reviewers for their insightful feedback. Regarding the question about whether Long COVID symptoms were the reason for re-admission in any of the readmitted patients, it's worth noting that while Long COVID symptoms were not documented as the principal or associated diagnosis in any of the discharge summaries/medical files, it is possible that some of the patients may be experiencing symptoms related to Long COVID at the time of readmission, but we did not find any documentation. We will incorporate this point in the manuscript.

**Comment 2:**

“On multivariable analysis, after adjusting for age, gender, co-morbidity, and disease severity, vaccination was found to be an independent predictor of long COVID with an Odds ratio of 2.42(95% CI 1.52-3.84) for the unvaccinated cohort”.
This could probably be rephrased to lack of vaccination was found to be an independent predictor of long COVID……….

**Reply :**

Thank you for the suggestion. The proposed rephrasing is more rational and we have incorporated this change in the revised manuscript using 'track changes’.

**Comment 3:**

3. “Around 22 patients (4.58%) died after discharge from the hospital, however, it was difficult for us to assess the cause of death through telephonic interviews.”
a) Could you ascertain the point of time at which these deaths occurred….after 6 months or a year after discharge?
b) And, were the patients contacted anytime in between or directly after a year ?
c) Were the patients following up elsewhere during the year ?
d) Was imaging of the chest performed on an OPD basis in any of the patients, to attribute the fatigue to post COVID sequelae like fibrosis ?

**Reply :**

The answers to comment 3 are given as follows:

a) Yes, we did examine the timing of these post-discharge deaths. The patients who expired following their discharge had a median survival duration of 31.5 days (IQR 7.5-213).
b) The patients were contacted and interviewed one year after their discharge from the hospital. We did not contact them in between.
c) While some patients may have sought follow-up care at other healthcare facilities during the year following their discharge, however, we did not include this query as part of our interview.
d) Around 28.3% (n=136) of patients have chest X-rays done in the OPD (at any point from their hospital discharge up to the one-year interview). Fibrosis and consolidation were observed in 8.7% (n=42) and 7.7% (n=37) of the patients respectively, the rest of the chest X-rays were reported as normal. However, this percentage is underestimated as many of the patients may have chest X-rays done outside of our healthcare system.

We have included these points in our manuscript and can be seen as track changes.

**Comment 4:**

4. Taken together, it was observed that patients with severe/critical disease and unvaccinated status had an increased risk of long COVID and higher readmission rates than those in the non-severe and vaccinated cohort.
The above statement gives the impression that higher readmission rates were due to long COVID 19

**Reply :**

I appreciate your observation, and I can see how the statement may have given that impression. We have removed this statement and provided clarity wherever we have mentioned readmissions.

**Comment 5:**

Reported data regarding post-COVID sequelae one year after discharge from the hospital are similar and showed the persistence of symptoms in COVID-19 survivors…please clarify this statement

**Reply :**

We wanted to say that the data from across the world also show similar results but this statement was removed while we were revising our discussion.

**Comment 6:**

The discussion does appear long. Can be made crisper and to the point to hold the reader’s attention.

**Reply :**

We understand your advice for a more concise and focused discussion. So as per your recommendation, we have rewritten the discussion to hold the reader's attention.
